# Effects of Switching From Another Sodium-Glucose Cotransporter 2 Inhibitor to Tofogliflozin on Nocturia in Patients With Type 2 Diabetes

**DOI:** 10.7759/cureus.59411

**Published:** 2024-04-30

**Authors:** Saori Inoue, Hiroko Yasuda, Kaoru Yoshida, Kazuaki Mori, Koichiro Ogawa, Yoko Yokotsuka, Hideki Okamoto

**Affiliations:** 1 Internal Medicine, Meitetsu Hospital, Nagoya, JPN; 2 Internal Medicine, Kakehashi Tonyobyo Kojyosen Clinic, Nagoya, JPN

**Keywords:** quality of life, tofogliflozin, nocturia, sodium-glucose cotransporter 2 inhibitor, type 2 diabetes

## Abstract

Objective: We aimed to characterize the effects of a switch from another sodium-glucose cotransporter 2 (SGLT2) inhibitor to tofogliflozin, which has a shorter half-life, in Japanese patients with type 2 diabetes. In particular, we aimed to assess the changes in the frequency of nocturnal urination and other parameters after four months of treatment.

Methods: A cohort of 31 patients who were taking SGLT2 inhibitors other than tofogliflozin was selected for a switch to tofogliflozin. After four months, their clinical parameters were assessed. In addition, questionnaires were administered to evaluate changes in the frequency of urination during the day, the amount of water intake, and the quality of sleep of the participants at this time point.

Results: Data for 30 of the participants were analyzed. We documented the following comorbid conditions of the urinary system among the participants: prostatic hypertrophy (4, 13%) and prostate cancer (1, 3.3%). The SGLT2 inhibitors that the participants had been using before switching to tofogliflozin were empagliflozin (16, 53%), dapagliflozin (4, 13%), canagliflozin (8, 27%), luseogliflozin (1, 3.3%), and ipragliflozin (1, 3.3%). There was a significant decrease in the frequency of nocturnal urination, from 2.6 ± 0.83 to 2.1 ± 1.3 times (P* *= 0.014). However, there were no significant changes in any of the other measured parameters from baseline. The questionnaire survey showed that 10 (33%) participants experienced improvements in sleep quality.

Conclusions: The switch from another SGLT2 inhibitor to tofogliflozin may reduce the frequency of nocturnal urination, implying that it may have a positive impact on the quality of life of patients with type 2 diabetes.

## Introduction

The treatment of diabetes can involve the use of various drugs. Sodium-glucose cotransporter 2 (SGLT2) inhibitors inhibit the reabsorption of glucose in the proximal tubules, thereby promoting urinary glucose excretion and having a hypoglycemic effect. In addition to reducing blood glucose concentration, SGLT2 inhibitors have been reported to have multiple effects, including the induction of weight loss, lowering of blood pressure, circulation of uric acid concentration, cardioprotection, renoprotection, and amelioration of nonalcoholic fatty liver disease [1]. However, despite these beneficial effects, it is important to note that an increase in the frequency of urination typically accompanies the use of these drugs.

According to the 2018 definition of the International Consultation on Incontinence Research Society, nocturia is defined as waking up two or more times during the night to urinate [2]. Nocturia can be associated with lower urinary tract disorders, such as overactive bladder or prostate enlargement, but polyuria is also a common cause [3]. In patients with diabetes, factors such as osmotic diuresis owing to hyperglycemia and the use of SGLT2 inhibitors cause nocturia. The prevalence of nocturia increases with age, and waking up to urinate more than twice a night has been suggested to increase the risk of fracture and mortality [4]. It also negatively affects the quality of life (QOL) of patients [5]. More than half of the patients with diabetes and sleep disorders report being awoken by the need to urinate [6]. Therefore, the choice of a drug that would be associated with less nocturia may be important.

In Japan, there are currently six SGLT2 inhibitors in use: ipragliflozin, dapagliflozin, luseogliflozin, tofogliflozin, canagliflozin, and empagliflozin. Many of these have a half-life of >10 hours, and tofogliflozin has the shortest half-life of 5.4 hours [7]. In a previously reported crossover study [8] that compared the use of ipragliflozin and tofogliflozin, the frequencies of urination between 00:00 and 06:00 during the administration period of tofogliflozin and ipragliflozin were 1.0 times (1.0-2.0 times) and 2.0 times (1.25-3.0 times), respectively. Thus, the use of tofogliflozin is associated with a significantly lower frequency of nocturnal urination. This study also showed that the area over the glucose curve of continuous glucose monitoring (CGM) up to three hours postprandially was significantly smaller after breakfast, lunch, and dinner during the administration of tofogliflozin. Additionally, in a previous crossover study, patients undergoing insulin therapy were administered tofogliflozin or ipragliflozin [9] and had their urinary glucose excretion and blood glucose fluctuations assessed. The latter was assessed using intermittently scanned CGM. During this study, the use of tofogliflozin was associated with significantly lower nocturnal urinary glucose excretion between 22:00 and 08:00 than ipragliflozin.

The results of these previous studies suggest that switching to tofogliflozin, which has a shorter half-life than other SGLT2 inhibitors, may ameliorate some of the patient’s clinical symptoms, including their frequency of nocturnal urination and sleep quality, without worsening their blood glucose control. Therefore, in the present study, we investigated the effects of a switch to tofogliflozin in patients already taking another SGLT2 inhibitor by assessing a range of clinical indices, the frequency of nocturnal urination, and sleep quality. In previous studies, insulin was used in all patients [8,9], but in this study, the effect on nocturia in patients with type 2 diabetes, including those not on insulin, was examined.

## Materials and methods

Participants

We recruited patients aged ≥ 20 years who had type 2 diabetes and were receiving outpatient care at the Endocrinology and Metabolism Department of Meitetsu Hospital in Japan. The eligibility criteria were as follows: (1) the use of an SGLT2 inhibitor other than tofogliflozin for at least the preceding month and (2) a frequency of nocturnal urination of two or more times per night. Changes in the medication of participants other than tofogliflozin during the study period were made at the discretion of the medical staff involved. The exclusion criteria were as follows: (1) pregnancy, potential pregnancy, or planned pregnancy during the study period; (2) severe pre-existing renal impairment or dialysis before the start of the study; (3) the discontinuation of tofogliflozin during the study; and (4) poor control of diabetes (HbA1c ≥ 9%).

Study protocol

All the participants provided their informed consent and initiated the administration of 20 mg of tofogliflozin daily in the morning for four months. They underwent blood sampling, weight measurements, and blood pressure assessments at the time of enrollment and four months later. The frequency of nocturnal urination of the participants was recorded at the time of enrollment and four months later. A questionnaire survey was conducted regarding the nocturnal and daytime frequencies of urination, fluid intake, and sleep quality of the participants four months later.

Measurements

The measurements that were made at baseline and four months later were as follows: hemoglobin A1c (HbA1c), body mass index (BMI), serum creatinine concentration, estimated glomerular filtration rate (eGFR), blood pressure, liver enzyme activities (aspartate aminotransferase [AST] and alanine aminotransferase [ALT]), serum lipid concentrations (low-density lipoprotein [LDL]-cholesterol, triglycerides, high-density lipoprotein [HDL]-cholesterol), and the frequency of nocturnal urination. In addition, the changes in the frequency of daytime urination, water intake, and sleep quality over the four months of the study were assessed.

Statistical analysis

All statistical analyses were conducted using SPSS v27.0.1. (IBM Corp., Armonk, NY). The data are presented as mean ± standard deviation, and comparisons between baseline and four months later were performed using the Shapiro-Wilk test to confirm the normal distribution of datasets, owing to the small sample size. The Wilcoxon test was used to compare non-normally distributed datasets, such as ALT activity and the frequency of nocturnal urination; and normally distributed datasets were compared using the paired t-test. The significance level was set at P < 0.05.

Research involving human participants

All the procedures followed were according to the ethical standards of the responsible committee on human experimentation and the Helsinki Declaration of 1964 and its amendments.

Informed consent

Written informed consent was obtained from all the participants. The Ethics Committee of the Meitetsu Hospital issued approval (No.: 247) on February 16, 2022.

## Results

A total of 31 participants were enrolled between March 1, 2022, and July 1, 2023. Data for 30 of these were included in the analysis because one participant discontinued the drug after two months owing to expressing a desire to stop taking it for well-controlled glycemia.

Participants were elderly with a mean age of 72 ± 9.2 years and a mean diabetes duration of 14 ± 11 years. They were non-obese with a mean BMI of 24 ± 4.4 kg/m^2^, had good glycemic control with HbA1c of 7.1% ± 0.83%, and maintained insulin secretion with serum C-peptide immunoreactivity of 2.6 ± 1.9ng/mL. Creatinine and urinary microalbumin levels were 0.86 ± 0.26 mg/dL (normal value: 0.46-0.79 mg/dL) and 65 ± 15 mg/g creatinine (normal value: 24.6 mg/g creatinine), respectively, indicating that the patients had preserved renal function but had advanced diabetic nephropathy (Table 1). We documented the following comorbid conditions of the urinary system among the participants: prostatic hypertrophy in four participants (13%) and prostate cancer in one participant (3.3%). The concomitant medications were antihypertensive drugs for 19 participants (63%), lipid-lowering drugs for 19 participants (63%), diuretics for four participants (13%), and medication to alleviate frequent urination for four participants (13%).

**Table 1:** Patient characteristics at baseline (n = 30) Data are expressed as mean ± SD or n (%). SGLT2: Sodium-glucose cotransporter 2; SD: Standard deviation; BMI: Body mass index.

		Baseline
Age (years)		72 ± 9.2
Sex (Male/Female), n (%)		22 (73)/8 (27)
HbA1c (%)		7.1 ± 0.83
Body weight (kg)		64 ± 15
BMI (kg/m^2^)		24 ± 4.4
Duration of diabetes (years)		14 ± 11
Serum C-peptide (ng/mL)		2.6 ± 1.9
Creatinine (mg/dL)		0.86 ± 0.26
Urinary microalbumin (mg/g creatinine)		65 ± 15
Urological diseases	Prostatic hypertrophy, n (%)	4 (13)
	Prostate cancer, n (%)	1 (3.3)
	Others, n (%)	1 (3.3)
Concomitant medications	Antihypertensive drugs, n (%)	19 (63)
	Lipid-lowering drugs, n (%)	19 (63)
	Diuretic, n (%)	4 (13)
	Medicine for frequent urination, n (%)	4 (13)
Antidiabetic drugs	Dipeptidyl peptidase 4 inhibitors, n (%)	17 (57)
	Metformin, n (%)	20 (67)
	Glinide, n (%)	7 (23)
	Sulfonylurea, n (%)	7 (23)
	Thiazolidine, n (%)	2 (6.7)
	α-glucosidase inhibitor, n (%)	5 (17)
	Imeglimin, n (%)	1 (3.3)
	Glucagon-like peptide-1 receptor agonist, n (%)	9 (30)
	Insulin, n (%)	4 (13)
Pre-treatment SGLT2 inhibitors	Empagliflozin, n (%)	16 (53)
	Dapagliflozin, n (%)	4 (13)
	Canagliflozin, n (%)	8 (27)
	Luseogliflozin, n (%)	1 (3.3)
	Ipragliflozin, n (%)	1 (3.3)

The most common concomitant medications for diabetes were dipeptidyl peptidase-4 (DPP-4) inhibitors (in 17 participants, 57%) and metformin (20, 67%). Insulin (4, 13%) was also included. The SGLT2 inhibitors that the participants had been using before switching to tofogliflozin were empagliflozin (16, 53%), dapagliflozin (4, 13%), canagliflozin (8, 27%), luseogliflozin (1, 3.3%), and ipragliflozin (1, 3.3%).

**Figure 1:**
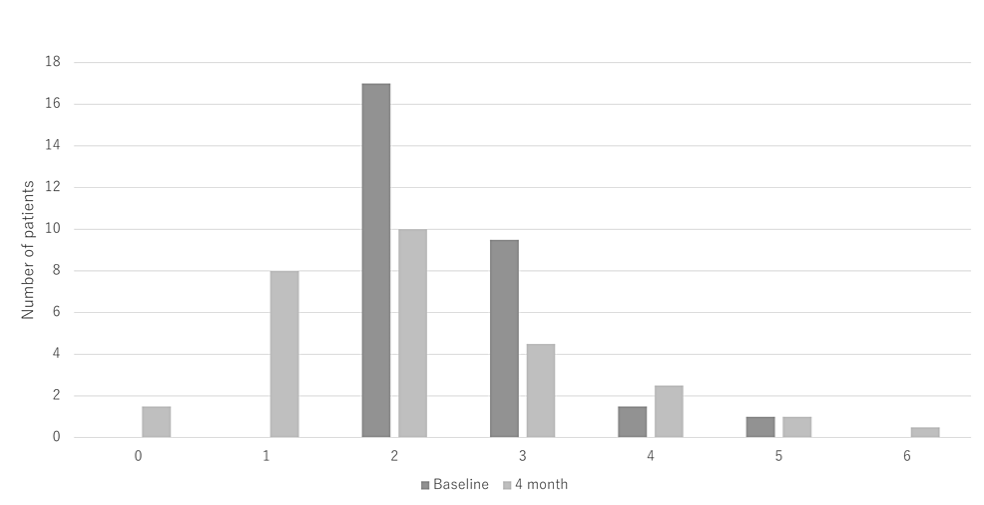
Histogram of nocturnal urination frequency

There were no significant changes in HbA1c, body weight, BMI, creatinine, eGFR, AST, ALT, blood pressure, LDL cholesterol, HDL cholesterol, or triglycerides during the four months of the study (P > 0.05). However, there was a significant decrease in the frequency of nocturnal urination from 2.6 ± 0.83 times to 2.1 ± 1.3 times (P＜0.05) (Table 2). A histogram of nocturnal urination frequency is presented in Figure 1.

**Table 2:** Efficacy of four months of additional tofogliflozin treatment in patients (n = 30) *P < 0.05 vs pre-treatment. Data are expressed as mean ± SD. SD: Standard deviation; BMI: Body mass index; eGFR: Estimated glomerular filtration rate; LDL: Low-density lipoprotein; HDL: High-density lipoprotein.

	Baseline	4 months later	P-value
HbA1c (%)	7.1 ± 0.83	7.2 ± 0.76	0.669
Body weight (kg)	64 ± 15	64 ± 16	0.765
BMI (kg/m^2^)	24 ± 4.4	24 ± 4.2	0.727
Creatinine (mg/dL)	0.86 ± 0.26	0.85 ± 0.29	0.644
eGFR (mL/min/1.73 m^2^)	66 ± 15	67 ± 18	0.623
AST (U/L)	19 ± 5.5	21 ± 7.2	0.134
ALT (U/L)	20 ± 10	22 ± 15	0.108
Systolic blood pressure (mmHg)	133 ± 18	136 ± 15	0.666
Diastolic blood pressure (mmHg)	69 ± 12	69 ± 11	0.435
LDL cholesterol (mg/dL)	105 ± 41	107 ± 31	0.364
HDL cholesterol (mg/dL)	56 ± 11	59 ± 13	0.063
Triglyceride (mg/dL)	137 ± 73	132 ± 66	0.671
Nocturnal urination frequency (times/night)	2.6 ± 0.83	2.1 ± 1.3	0.014*

The results of the questionnaire survey are summarized in Table 3. The frequency of daytime urination did not change in 25 participants (83%), and water intake did not change in 19 participants (63%) but was increased in eight participants (27%). Sleep quality did not change in 18 participants (60%), but improved in 10 participants (33%).

**Table 3:** Questionnaire on urination and sleep compared to four months ago (n = 30)

Type of question		n (%)		n (%)		n (%)
Frequency of urination during the day	Increase	1 (3.3)	No change	25 (83)	Decrease	4 (13)
Water intake	Increase	8 (27)	No change	19 (63)	Decrease	3 (10)
Sleep quality	Improvement	10 (33)	No change	18 (60)	Worsening	2 (6.6)

In terms of safety, one participant was hospitalized owing to ileus during the study, but after their condition improved, they resumed tofogliflozin use. In one participant, the frequency of nocturnal urination became unmeasurable because they stopped using the toilet for nighttime urination.

## Discussion

We have shown that patients experience a reduction in the frequency of nocturnal urination when they switch to tofogliflozin from another SGLT2 inhibitor. It has previously been shown that urinary glucose excretion and urine volume correlate [10]. Tofogliflozin presumably reduces the frequency of nocturnal urination because of its shorter half-life in circulation. Nocturia has been suggested to affect the QOL of patients [5], and the questionnaire survey showed that 10 participants (33%) experienced improvements in sleep quality. While it is notable that 33% of participants reported improvement, it is important to note that many individuals did not experience a change. However, this study represents a one-time data collection and lacks statistical analysis. Therefore, while there is a suggestion that switching to tofogliflozin may improve sleep quality, further research is needed with a control group for comparison. This highlights an area for future investigation and study.

Tofogliflozin exhibits a 2900-fold selectivity for SGLT2 over SGLT1, which is higher compared to other SGLT2 inhibitors such as luseogliflozin (1600-fold), empagliflozin (1100-fold), ipragliflozin (860-fold), dapagliflozin (610-fold), and canagliflozin (290-fold) [11].

The protein-binding rate of tofogliflozin was 83%, whereas that of ipragliflozin was between 94.6% and 96.5%. In the blood, these drugs are bound to plasma proteins such as albumin. The relatively low protein-binding rate of tofogliflozin indicates that comparatively more tofogliflozin is filtered through the glomerulus and reaches the renal tubules, the site of action of SGLT2 inhibitors [9].

Its shorter half-life, low protein-binding rate, and higher selectivity for SGLT2 suggest a stronger urinary glucose excretion effect during the day compared to other SGLT2 inhibitors, with potential attenuation of effects during the night. In a previous study, tofogliflozin administration was shown to be associated with significantly higher time in range (TIR) (blood glucose level 70-180 mg/dl) per day for CGM than ipragliflozin, and there was also greater urinary glucose excretion between 08:00 and 22:00 [9]. The urinary glucose excretion and glucose-lowering effects of these drugs correlate [10]. Therefore, compared to ipragliflozin with its longer half-life, it is likely that tofogliflozin use is associated with greater urinary glucose excretion and glucose-lowering effects during the day, when patients’ blood glucose concentrations tend to be high, following meals. In the present study, the glycemic control of the participants was evaluated based on HbA1c alone, and no significant changes were observed. Thus, the switch to tofogliflozin, with its shorter half-life, did not adversely affect glycemic control, and there were no hypoglycemic events.

SGLT2 inhibitors have demonstrated protective effects on the heart and kidneys [12-14]. In trials comparing tofogliflozin with other SGLT2 inhibitors, no significant differences were observed in their impact on cardiac and renal functions [15,16]. From the results of the aforementioned studies, the cardiorenal protective effects are considered a class effect of SGLT2 inhibitors. In this study, although the duration was relatively short, no significant changes were observed in renal function. Evaluation of parameters related to the heart has not been conducted.

Although the present study lasted a relatively short period, the participants experienced no significant weight loss following the switch. This characteristic may imply that this drug is a useful therapeutic option for older individuals who have been at risk of frailty. However, it is worth noting that, as for other SGLT2 inhibitors, tofogliflozin has been reported to have weight-reducing effects [17]. Therefore, caution should still be exercised regarding its use in individuals at risk of frailty.

The factors implicated in nocturia include not only polyuria but also sleep disturbances and a reduction in bladder capacity. Given the large number of older participants in the present study, there may have been multiple contributors to the frequency of nocturnal urination. Some of the participants showed a poor response to tofogliflozin, and further subgroup analyses, considering factors such as the underlying causes of frequent urination and the presence of comorbidities such as prostate enlargement, are necessary to further understand the causes of such differences in response. In this study, due to insufficient sample size, subgroup analysis could not be conducted, thus there is a need for a study with an increased sample size.

The limitations of this study include the small sample size and its single-center design. Additionally, we only studied patients who frequently urinated (at least twice) at night, and the study was not blinded. While there is a general tendency for an increase in the frequency of urination during winter, we recruited participants throughout the year in the present study, making it challenging to exclude seasonal variations. To validate the present findings and ensure their alignment with real-world outcomes, larger, long-term, multicenter, blinded studies should be performed in the future. Finally, the assessment of the safety of the switch in medication was limited by the administration of tofogliflozin for only four months. Although dehydration, urinary tract infections, and skin disorders were not recorded during the present study, caution is advised regarding using tofogliflozin, as for other SGLT2 inhibitors.

## Conclusions

Results of a single-center, prospective study conducted with type 2 diabetic patients with two or more nocturia as subjects showed that switching from other SGLT2 inhibitors to tofogliflozin reduced the nocturnal voiding frequency. A follow-up questionnaire administered four months later suggested a possible improvement in the QOL. However, further large comparative studies are needed to corroborate the results of this pilot study.

SGLT2 inhibitors are renal and cardioprotective and are useful drugs in the treatment of diabetes. A variety of SGLT2 inhibitors are commercially available, and the possibility that tofogliflozin may reduce urinary frequency, a side effect of SGLT2 inhibitors, may influence clinicians' drug selection.
